# Negative and positive self-beliefs in social anxiety: The strength of believing mediates the affective response

**DOI:** 10.1371/journal.pone.0281387

**Published:** 2023-03-15

**Authors:** Sabrina Golde, Sophie Ludwig, Sven Lippoldt, Jérôme Rimpel, Lars Schulze, Matthias Haucke, Babette Renneberg, Stephan Heinzel

**Affiliations:** 1 Department of Education and Psychology, Clinical Psychology and Psychotherapy, Freie Universität Berlin, Berlin, Germany; 2 Department of Clinical Psychology and Neuropsychology, Institute of Psychology, Johannes Gutenberg-University Mainz, Mainz, Germany; 3 Klinik für Psychiatrie und Psychotherapie, Charité –Universitätsmedizin Berlin, Berlin, Germany; University of Leipzig, GERMANY

## Abstract

**Background and objectives:**

Current cognitive models of social anxiety disorder (SAD) propose that individual, situation-specific self-beliefs are central to SAD. However, the role of differences in the degree to which individuals with social anxiety are convinced of self-beliefs, in particular positive ones, is still not fully understood. We compared how much high and low socially anxious individuals agree with their own negative and positive self-beliefs. Furthermore, we investigated whether agreeing with one’s self-belief can explain the relation between negative affect in response to self-beliefs and social anxiety. Specifically, we were interested whether social anxiety increases negative affect in response to self-beliefs through an increase in agreement.

**Methods:**

We developed a new experimental self-belief task containing positive and negative semi-idiosyncratic, situation specific self-beliefs typical of high social anxiety and included a direct measure of agreement with these beliefs. Using extreme group sampling, we a-priori selected high (n = 51) and low (n = 50) socially anxious individuals. By multi-level mediation analysis, we analyzed agreement with self-beliefs in both groups and its association with affect.

**Results:**

High and low socially anxious individuals chose similar self-beliefs. However, high socially anxious individuals (HSA) agreed more with negative self-beliefs and less with positive self-beliefs compared to low socially anxious individuals (LSA). HSA individuals reported increased negative affect after both, exposition to negative and positive self-beliefs compared to LSA. We found that social anxiety increases affective responses towards negative-self beliefs through an increase in agreeing with these self-beliefs.

**Conclusions:**

These findings suggest that cognitive models of social anxiety can be improved by including not only the content of a self-belief but also the strength of such a belief. In addition, they emphasize the relevance of positive self-beliefs in social anxiety, which has frequently been overlooked.

## Introduction

Social anxiety disorder (SAD) is one of the most frequent anxiety disorders [1, 2], with a high burden of disease and an often chronic course [3]. Cognitive models of social anxiety [4–6] are central to understanding and treating social anxiety disorder (SAD). These models are based on the idea that individual negative beliefs about oneself (e.g. “I am unacceptable”) are at the core of the disorder. These negative beliefs are triggered in social situations, which makes these social situations appear threatening to the individuals. As a result, the individuals experience anxiety symptoms [4, 6, 7]. Negative self-beliefs can be defined as a negatively biased and maladaptive view or belief about the social self (either conditionally or unconditionally) or a negatively biased approach to social situations. In turn, positive self-beliefs can be defined as a positive and adaptive view or belief about the social self or an optimistic approach to social situations. Despite its apparent importance for our understanding and treatment of SAD, we know relatively little about the relationship between agreement with self-beliefs of different valence and the experience of social anxiety in (non-clinical) individuals. Thus, the main aim of the current study was to examine the role of agreement with situation-specific,–positive and negative–self-beliefs for the experience of social anxiety. Furthermore, we sought to analyze the relationship between agreement in self-beliefs and the individual’s affective response.

Spontaneous negative beliefs about one’s competence, likability or attractiveness in social situations seem to be part of normal experience. Unwanted intrusive doubts are at least to some degree a common and normative experience [8]. However, the degree to which people agree with these negative thoughts varies significantly. Agreement with negative self-beliefs is an important factor that underpins cognitive models of social anxiety, however, the degree to which the role of the degree to which individuals agree is still not fully understood [4, 6, 7]. Clinically, however, cognitive treatment of SAD has been focusing on testing these negative self-beliefs and tracking agreement with them continuously [9].

The role of positive self-beliefs in social anxiety has received relatively little empirical attention to date. There has, however, been some studies examining the role of processing positive information in general (e.g., praise) [10]. Findings so far suggest that people with SAD show a downward bias when recalling positive feedback, that is they recall positive feedback more negative than it has been [11]. Also, CBT leads to a reduction in self-reported negative affect when reacting to social praise [12] well as decreased fear of positive evaluation [10]. Moreover, research on self-compassion suggests that self-kindness might be linked to lower social anxiety and less associated symptoms [13–17]. Lastly, there are two studies that found positive self-views to be associated with symptom reductions in SAD [18, 19]. Taken together, positive self-beliefs and, more specifically, a sense of conviction in positive self-views might be linked to lower social anxiety in individuals. Since studies exploring this assumption are rare, the relationship between agreement with positive self-beliefs and the experience of social anxiety in individuals is a central aim of this study.

Regarding agreement with negative self-beliefs, there have been some studies supporting its importance for SAD. Most of them have used self-reports, e.g. the Core Beliefs Questionnaire [20]. Results indicated that self-reported high agreement with negative self-beliefs was associated with higher SAD symptom severity. Symptom reduction after CBT was associated with less agreement with negative self-beliefs [9, 21, 22]. Because the validity of self-report measures is often impeded by response biases such as impression management, memory bias or socially desirable responding [23], we investigated agreement on self-beliefs in an experimental study.

The most common experimental task to assess self-beliefs in general is the “self-referential-encoding task” (SRET [24]). Participants indicated their agreement to standardized adjectives (e.g. “likeable”, “boring”). This agreement was used as a proxy for self-beliefs. A dichotomous answering scale (“yes” or “no”) and reaction time measures are used. Results indicated that patients with SAD endorsed more negative and less positive self-referential adjectives than healthy controls [18, 25]. Also, CBT increased positive and decreased negative self-views. Interestingly, only the increase in positive self-views by CBT had an effect on social anxiety symptoms [18, 19].

The SRET has several theoretical and methodological shortcomings: First and foremost, the SRET uses standardized adjectives across situations. However, cognitive models of SAD assume idiosyncratic self-beliefs that are activated by specific social situations in contrast to more global and stable ones often found in other mental disorders such as depression [4]. Second, the dichotomous answering scale might further increase response biases, such as dis-acquiescence, i.e., respondents are more likely to disagree or give a negative response. This might thus further increase differences between groups. Third, one could argue that the self-concept as it is assessed by the SRET or questionnaires represents a more global top-down self-assessment. In contrast, specific core beliefs are triggered bottom-up in specific social situations [4, 26]. In both studies the term “self-view” instead of self-belief is used [18, 19]. Furthermore, the SRET does not assess the effect of those adjectives on affect. However, this link seems to be important to better understand the association between self-beliefs in social anxiety and emotional well-being.

Only two studies experimentally examined the role of individual self-beliefs in SAD [27, 28]. Only the latter one included positive self-beliefs. Importantly, none of them has measured the degree to which participants agreed with these self-beliefs. In the task developed by Goldin and colleagues [27] participants had to write a paragraph about autobiographical social situations involving social anxiety, humiliation or embarrassment. The experimenters then derived negative self-beliefs based on those paragraphs. Standardized neutral self-relevant statements were included for comparison. Results showed that patients with SAD reported higher negative emotions than healthy controls (HC), when confronted with negative self-beliefs [27]. In the task by Hofmann and colleagues [28] participants recorded all their thoughts while they were anticipating a social stress test, pre and post CBT. Results showed a significant reduction of negative thoughts in the CBT group compared to waitlist controls. Reduction of negative thoughts significantly correlated with social anxiety symptom reduction; no changes were found for positive thoughts. However, this experimental approach required experimenters to rate more than 2000 individual thoughts, implying a low feasibility.

In general, while these studies in clinical populations might assume that individuals strongly believe their negative thoughts, explicit measurements of agreements with self-beliefs are mostly missing. There is one study by Tanner et al. [26] in individuals without a mental disorder that measured agreement with negative self-beliefs related to a forthcoming social threat task (giving a speech) using the Social Cognitions Questionnaire (SCQ). In this study, highly socially anxious individuals agreed more strongly with their negative self-beliefs compared to low socially anxious individuals but did not assess affective consequences.

To summarize: Idiosyncratic self-beliefs activated by specific social situations are assumed to be at the core of social anxiety. Social anxiety has been linked to higher agreement with negative self-beliefs and there is initial evidence for less positive self-beliefs in social anxiety [e.g., 18, 20, 22]. In addition, increased negative and diminished positive affect in social anxiety is a consistent finding, even when controlling for depressive symptoms (for a review see Dryman et al., 2018 [29]). There is a lack of studies experimentally examining the role of agreement with positive self-beliefs. To the authors’ knowledge, the effect of agreeing with situation-specific individual self-beliefs on affect in social anxiety has not yet been examined.

Based on previous research, we developed a new self-belief task, including positive as well as negative semi-idiosyncratic, situation-specific self-beliefs typical of high social anxiety. To increase external validity, we adapted the task to each participants’ personally relevant social situations. The task thus allows to expose participants to their individually relevant self-beliefs in specific social situations and to assess their corresponding affective response on a trial level. To quantify the strength and relevancy of the self-beliefs, we included a direct measure of agreement with the belief. The task thus combines an idiosyncratic yet standardized and controlled approach to analyze the relationship between the strength of a self-belief and affect in individually relevant social situations.

We investigate the following four hypothesis: First, high socially anxious individuals report higher negative affect and lower positive affect following exposition to negative and positive self-beliefs compared to low-socially anxious individuals. Second, high socially anxious individuals agree more strongly with negative self-beliefs and less strongly with positive self-beliefs compared to low socially anxious individuals. Third, a high agreement with a negative or positive self-belief is associated with an increased subjective affect rating. Fourth, we hypothesize that the association between social anxiety and affect is partially mediated by the agreement with the self-belief. That is, the stronger the conviction that the belief is true, the stronger its association affect.

## Method

### Participants

We recruited participants through public advertisement on local bulletin boards, mailing lists at the university and on social media. In a first step, we used an online screening procedure (Unipark, QuestBack GmbH, version EFS Fall 2017) to screen for eligibility. The screening comprised the German version of the social phobia inventory [SPIN; 30] to define either low or high social anxiety. To screen for possible comorbid mental disorders, we used the SCID-I (Structured Clinical Interview for DSM-IV) screening items, including the first two items for affective disorders. In addition, we used the SCID-II items on borderline and antisocial personality disorder [31] to exclude participants with major difficulties in emotion regulation, impulse control or identity. Additionally, we asked participants to indicate whether they suffered from any past or current neurological condition (e.g., epilepsy) or major somatic illness (e.g. cardiovascular disease).

Low socially anxious (LSA) participants were included, if they displayed a SPIN score < 15 and did not screen positive for any comorbid mental, neurological or major somatic disorder. This was chosen as a cut-off since a SPIN score below 15 can be considered below average levels of social anxiety levels found in psychologically healthy individuals (Connor et al., 2000; Sosic, Gieler, and Stangier 2008). We included high socially anxious (HSA) participants, if they displayed a SPIN score > 25. This cut-off was recommended by [32] for clinical comparison studies. We did not exclude HSA participants, if they screened positive for another anxiety or mood disorder. Comorbid anxiety or mood disorders are common in social anxiety [3].

**Fig 1:**
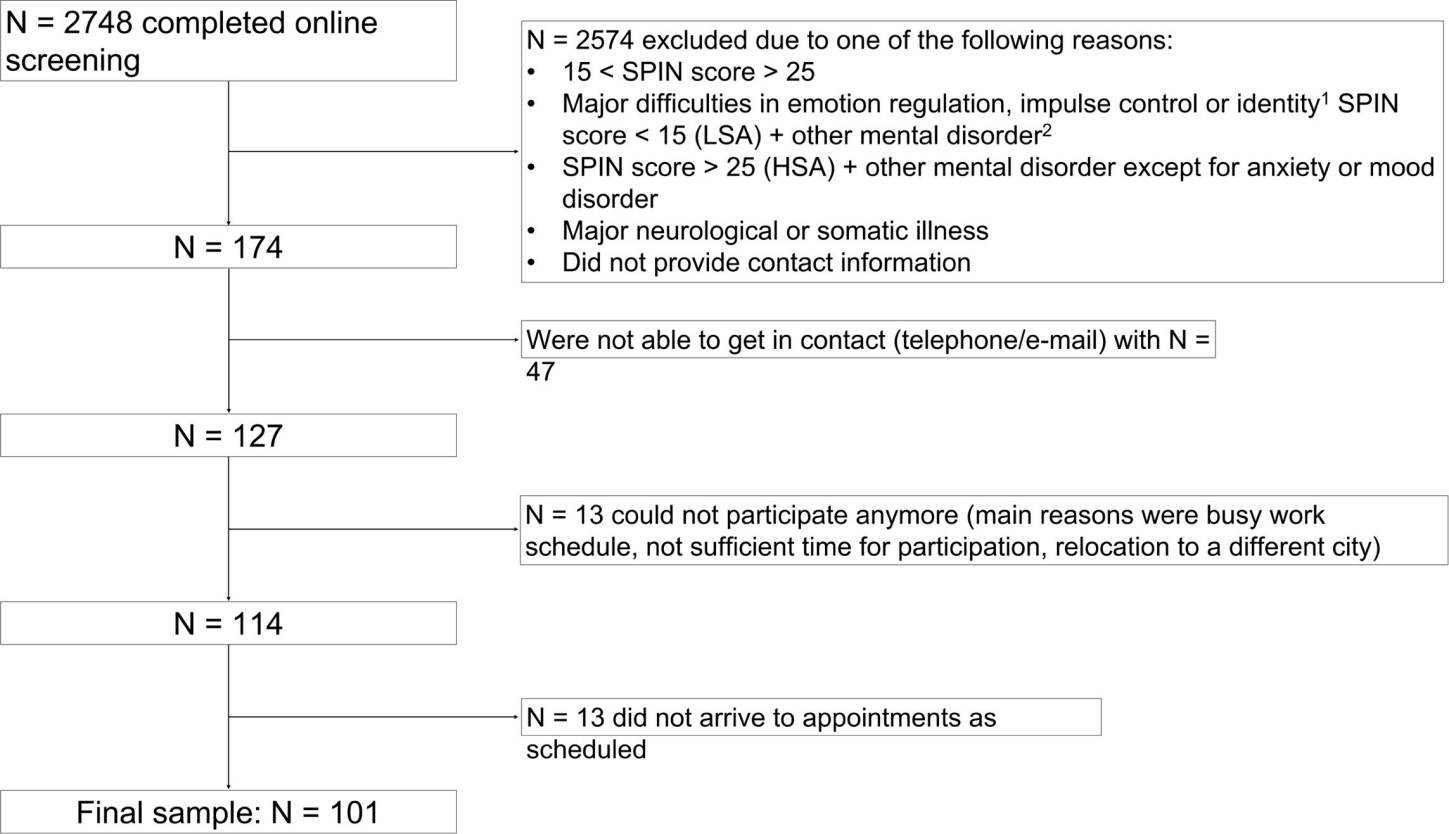
Flowchart of participant selection. ^1^ assessed by SCID-II items on borderline and antisocial personality disorder; ^2^assessed by SCID-I screening items. SPIN = social phobia inventory; SCID = Structured Clinical Interview for DSM-IV; LSA = low social anxiety; HAS = high social anxiety.

A total of N = 2748 completed the online screening. N = 174 individuals met our inclusion criteria and provided contact information. We were able to contact N = 127 eligible participants via telephone and made appointments with N = 114 individuals. N_HSA_ = 51 and N_LSA_ = 50 participants arrived as scheduled. See Fig 1 for a flowchart of participant selection. The data assessment was part of a larger research project, including online assessment and one lab session with several tasks and concurrent EEG measurements. All participants provided written informed consent and were recompensed with either 40€ or credit points. The study was approved by the ethics committee of Freie Universität Berlin (159/2017).

### Measures

#### Social Phobia Inventory [30, SPIN; 33]

We used the SPIN to assess social anxiety symptom severity. The SPIN is a 17-item screening instrument that assesses fear and avoidance of social situations as well as physiological symptoms of anxiety on a five-point Likert scale. For the German version a cut-off score of > 25 for social anxiety disorder has been established using a representative sample [32], thus we used a cut-off of > 25 for the HSA group. We chose the mean SPIN score (M = 15) of this representative sample as a cut-off score for low social anxiety, indicating average or below average social anxiety symptom severity.

### Self-belief-task

#### Stimuli development

To generate the stimuli set, we conducted a separate online-based study. We first identified n = 157 negative and n = 34 positive self-beliefs based on scales and questionnaires assessing self-beliefs in social anxiety (Supplement A for an overview of the scales in S1 File). Next, we selected n = 32 negative and n = 37 positive self-beliefs (n = 3 items were additionally constructed by reformulating negative items) based on expert’s ratings on item lengths, specificity for SAD and applicability for multiple social situations. Clark [34] distinguishes between conditional and unconditional beliefs about the self. To cover a wide range of possible negative and positive beliefs, we included conditional self-beliefs (e.g. “It will be embarrassing, if the others see me blush”), unconditional self-beliefs (“I am incompetent”) and negative outcome expectancies (“What I say, will sound stupid”) in this online study. Additionally, we constructed n = 55 neutral self-beliefs, using statements with self-reference (e.g. “I”, “my”) but no social aspects (e.g. “I like looking out of the window”).

We used this item-pool to conduct an independent, online-based pilot survey with a separate sample of n = 141 HSA participants (i.e. SPIN score > 25, mean age = 27.14, SD age = 9.25, 72.3% female, 77.4% high school diploma) and n = 82 LSA participants (i.e. SPIN score < 15, mean age 30.62, SD age = 11.08, 73.5% female, 87.8% high school diploma). Participants rated their agreement with and their affective response to these pre-selected self-beliefs. Based on the ratings, we selected those items that showed a significant mean difference between groups, resulting in n = 29 negative and n = 35 positive self-beliefs. An English version of these items can be found online (https://osf.io/w6xuy/). Additionally, we included a neutral condition with n = 20 statements specific to five neutral situations (e.g. “being at home”), that did not show a significant mean difference between groups.

#### Procedure before task

Participants first developed a fear hierarchy of their five personally relevant, most fear-inducing social situations in their lives (e.g. asking strangers for directions, giving a presentation). Participants also indicated the degree of fear for each situation on a scale between 0 = no fear at all, 50 = medium level, 100 = maximum level of fear. Next, participants assigned their most relevant four negative and four positive self-beliefs from the item-pool (developed in the pilot study described above) to each of the personal five negative social situations. The same self-beliefs could be used for two different situations. Neutral self-beliefs were not individually selected. An individual stimuli-set was therefore composed of 5 x 4 semi-idiosyncratic negative, 5 x 4 semi-idiosyncratic positive and 5 x 4 neutral self-beliefs. After selecting the self-beliefs, participants rated how much they agreed with each one on a visual analogue scale (from 0 to 250, with increment steps of 1). As scale anchors we used the portrait version of the 9-point self-assessment manikin (SAM) scale [35, 36].

#### Experimental task

**Fig 2:**
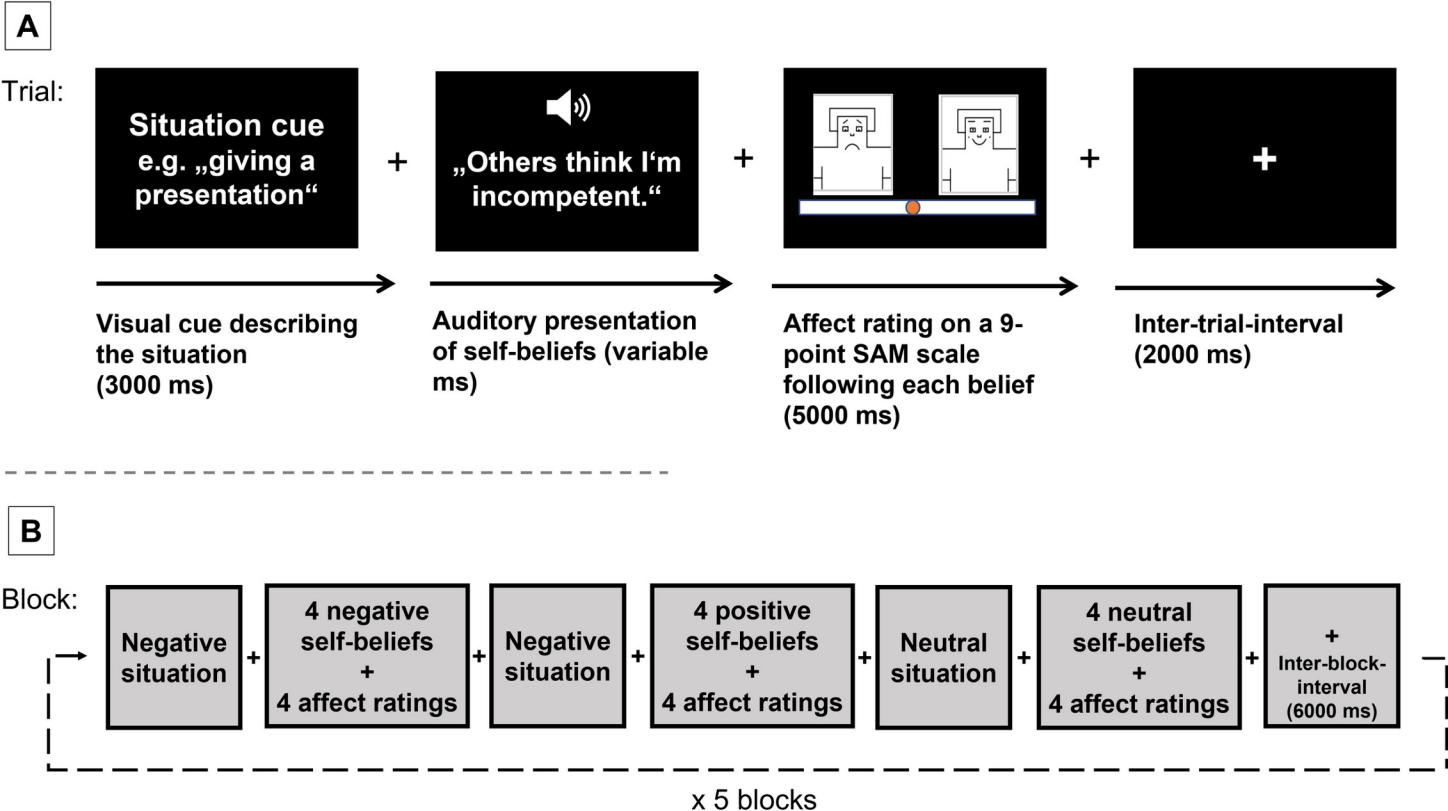
The self-belief task. (A) Example trial, starting with a visual cue describing an individual negative situation, next a negative self-belief was presented via loudspeakers. After listening to the self-belief, participants rated their affective response on a 9-point SAM scale. Each trial ended with a fixation cross. (B) The setup of a block. Each block started with a negative situation cue, followed by four negative self-beliefs. Next, the same negative situation cue was followed by four positive self-beliefs. Finally, a visual cue of a neutral situation and four neutral self-beliefs were presented. Participants rated their affective response after each belief. Each block ended with a fixation cross. In total, there were five blocks, using five negative and five neutral situations.

The self-belief task depicted in Fig 2 consisted of the five personally feared social situations (see above) and five predefined neutral situations. Participants were first instructed to vividly imagine themselves in the middle of the respective social situations. We developed and then tested the imagining of socially feared situations in a pilot sample by interviewing participants afterwards. The detailed procedure of the main task developed was as follows:

1. After a visual cue (3000 ms) describing the first negative situation (e.g. “giving a presentation”), the first negative self-belief was presented via loudspeakers (e.g. “others think I’m incompetent”) while a fixation cross was shown (variable ms). After listening, participants rated their affective response (5000 ms) on a visual analogue scale described above. All four negative self-beliefs, which the participant had previously assigned to his or her personal negative situation, were presented and the participants rated their affective response after each self-belief presentation (negative condition).
2. Again the visual cue of the negative situation (e.g. “giving a presentation”) was presented but followed by the four individual positive self-beliefs. After listening to one of the self-beliefs, participants rated their affective response (positive condition).
3. A visual cue of the first neutral situation was presented, followed by the four predefined neutral self-beliefs and a rating of the affective response (neutral condition).

This procedure was subsequently repeated for each of the five personal fear situations and five neutral situations. Thus, every block consisted of three (two times the negative situation + one time the neutral situation) times four trials; that is 12 trials per block and 60 trials in total. One trial consists of the auditory confrontation with a self-belief and the rating of one’s affective response. The situation with the highest anxiety level was always presented first and self-beliefs were kept in the same order as the participants had assigned them to the situations. The presentation of neutral trials was kept constant across participants. Every trial ended with the presentation of a fixation cross (2000 ms between trials and 6000 ms between blocks). 6000 ms between blocks was added to avoid spill-over effects between blocks.

We implemented a block-wise design, with self-beliefs being presented in blocks of four for each situation in the same order the participants had assigned them to each situation. This way, we were able to create a more realistic scenario where participants stay in the respective “mind-set”, thereby increasing ecological validity.

We used audio recordings of the self-beliefs to ensure a standardized stimuli-offset for all participants. The audio recordings were created using a text to speech plugin. Male participants had a male voice and female participants a female voice.

The experiment was programmed using Presentation (Version 20.0; Neurobehavioral Systems, Inc.) with a running time of ca 30 minutes, including 10–20 minutes for the preparation of the task (i.e. generation of situations, selection of self-beliefs, agreement ratings), and a short practice trial.

### Statistical analysis

To compare the selection of negative and positive self-belief statements between HSA and LSA, we first calculated the frequency with which each statement was chosen from our pre-selected item-pool by HSA and LSA individuals. We then ranked the negative and positive statements according to frequency for both groups separately. Subsequently, we calculated a Pearson correlation between the ranks of negative statements for HSA individuals and LSA individuals. Equivalently, we correlated the ranks of positive statements for HSA and LSA individuals.

We used a multilevel mediation modeling approach. The affective response ratings (level 1) were additionally nested in the statements (level 2), because each participant chose their individually most relevant set of statements. Contrast coding was used to compute main effects for social anxiety (HSA vs. LSA) and condition (negative, neutral, positive self-beliefs). Level 1 variables were not centered. All data were analyzed using R (R Core Team, 2019) with the following packages “lme4” [37] and “mediation” [38]. All data and analyses including a translated stimuli-set can be found online (https://osf.io/w6xuy/). Model description can be found in Supplement B in S1 File.

#### Mediation

**Fig 3:**
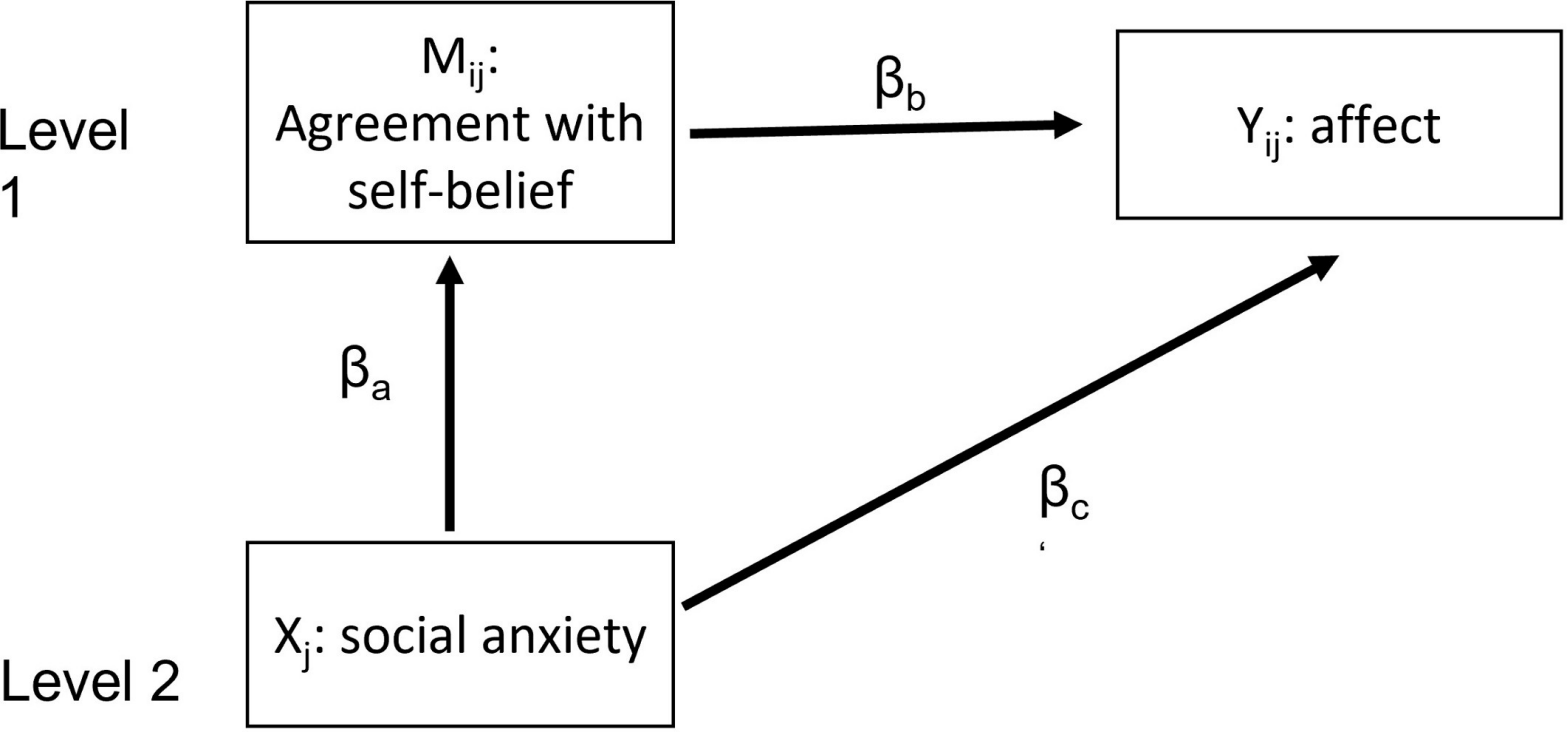
Schematic for the proposed multilevel mediation model. 2-1-1 mediation model, with social anxiety status (HSA vs. LSA) as a level two predictor, agreement with self-beliefs as a level one mediator and affect (either positive or negative) as a level one outcome variable. Mij = mediator, Xj = predictor, Yij = outcome, βc = direct effect, βaβb = mediated or indirect effect of social anxiety status on affective response.

We used a 2-1-1 design (s. Fig 3), with a level 2 predictor (social anxiety (HSA vs. LSA); X) affecting a level 1 mediator (M; agreement) and a level 1 outcome variable (Y; affect). For the model (M1), we used the traditional multilevel mediation method described by Krull and MacKinnon [39] for a 2-1-1 design, because it can easily be applied to a two- random-intercepts-model. However, following this approach, the estimations of the between group indirect effect might be biased [40]. We therefore used the “mediate” function from the R package “mediation” [38] to conduct a second mediation analysis for the basic model (M0) to double check our results [38, 41]. A description of the “mediate” package can be found in Supplement B in S1 File.

## Results

### Sample

Table 1 shows the sample’s demographic characteristics (N = 101). Participants were between 18 and 59 years old and had on average 12 years of education. 60% of participants were female. Participants did not differ in age, gender, years of education or proportion of students (49% vs. 40%) between groups (Table 1 in S1 File).

**Table 1:** Demographic sample characteristics.

	HSA	LSA	test statistics
SPIN (M, SD)	36.20 (7.99)	8.66 (3.95)	t = 22.02***, df^1^ = 73
age (M, SD)	28.78 (7.81)	29.62 (9.69)	t = -0.48, df = 94
number of women	34	33	χ^2^ = 2.00, df = 3
years of education (M, SD)	12.33 (2.05)	12.20 (1.75)	t = 0.35, df = 97
number of students	25	20	χ^2^ = 0.50, df = 1

*Note*. HSA = high social anxiety; LSA = low social anxiety; SPIN = Social Phobia Inventory; M = mean; SD = standard deviation,

^1^ we used Welch’s two sample t-test;

*p< 0.05,

p< 0.01**,

p < 0.001***.

Groups differed significantly in social anxiety symptoms. SPIN scores showed an excellent internal consistency (Cronbach’s α = .96) with scores ranging from 0 to 15 in the LSA group (M = 8.66, SD = 3.95) and from 25 to 60 in the HSA group (M = 36.20, SD = 7.99). The mean SPIN score in the HSA group is comparable to scores in a German speaking, treatment seeking population (M = 36.21, SD = 13.61) diagnosed with SAD [32].

### Self-belief task

Regarding affect ratings, results showed that HSA participants reported higher negative affect across self-belief conditions (significant main effect for social anxiety in the two random intercept models t(99) = -4.15, p < 0.001). Also, affect was generally lower for negative self-beliefs (compared to positive and neutral self-beliefs; t(3534) = -61.1, p < = 0.0001).

**Fig 4:**
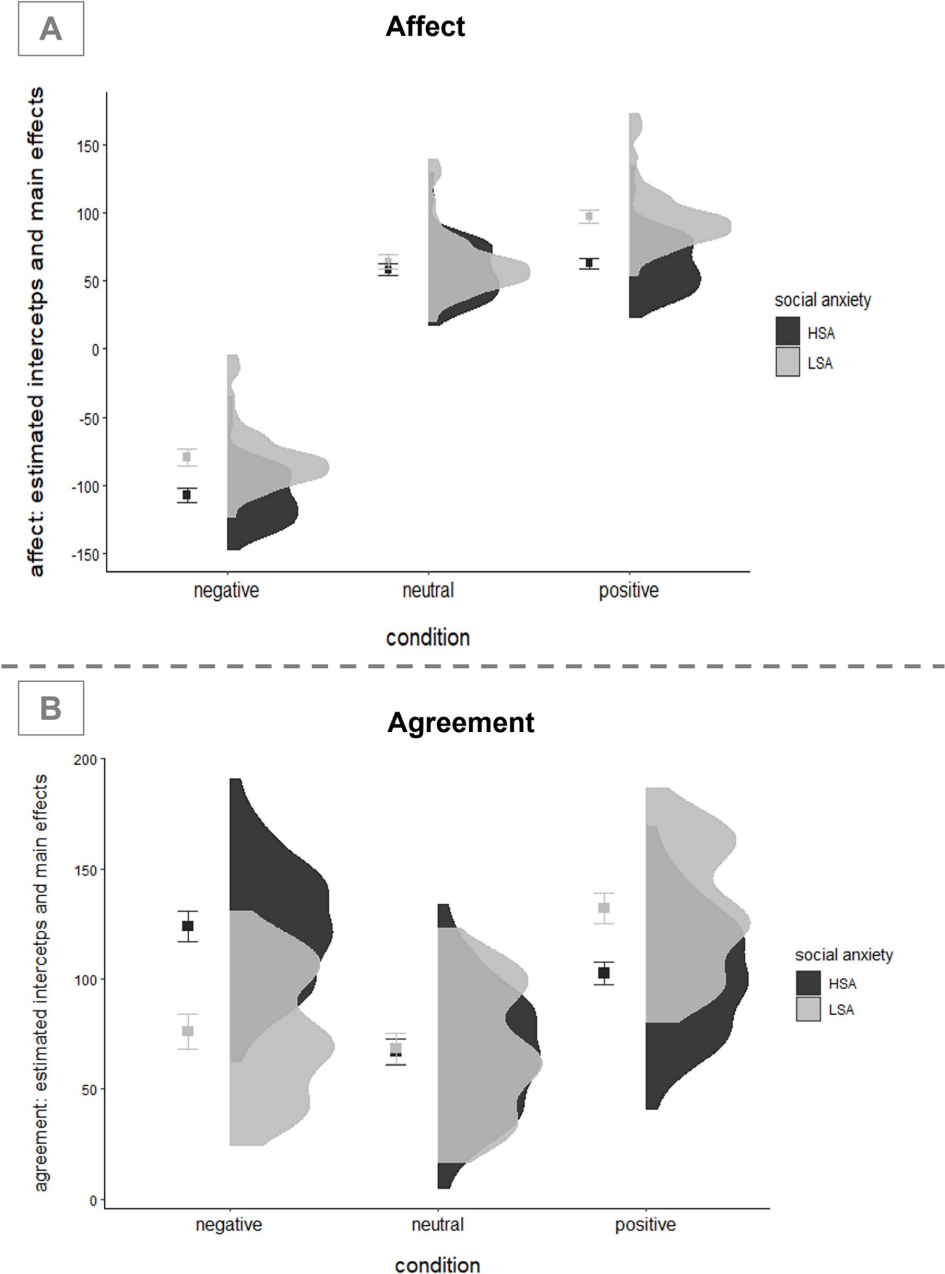
Affect and agreement ratings for negative, neutral, and positive self-beliefs. (A) Affect ratings. For each self-belief condition on the left-hand side: estimated mean affect ratings for high (HSA) and low (LSA) socially anxious participants; error bars represent estimated standard errors. Right hand side: distribution of estimated intercepts of affect ratings. (B) Agreement ratings. For each self-belief condition on the left-hand side: estimated mean agreement ratings for high (HSA) and low (LSA) socially anxious participants; error bars represent estimated standard errors. Right hand side: distribution of estimated intercepts of agreement ratings.

Moreover, there was a significant social anxiety x affect interaction (t(5933) = 5.57, p < 0.0001). Post-hoc comparisons with Bonferroni correction revealed that HSA participants reported higher negative affect both, in the negative (t = -4.41, p < 0.0001) and positive (t = -5.53, p < 0.0001) self-belief conditions compared to LSA participants. There was no difference between groups in the neutral condition (t = -0.93, p = 0.354). We will therefore focus on the affective response following exposure to positive and negative self-beliefs. Fig 4A shows the estimated mean affect ratings for each condition and the distribution of individual intercepts as estimated by model M1.

Fig 4B shows the estimated mean agreement ratings for each condition and the distribution of individual intercepts as estimated by model M1. There was a significant social anxiety x condition interaction (t(5931) = 10.39, p < 0.0001). Post-hoc comparisons with Bonferroni correction revealed that HSA participants reported higher agreement with negative self-beliefs (t = 5.99, p < = 0.0001) and less agreement with positive self-beliefs (t = -3.70, p < 0.0001) compared to LSA participants. There was no group difference in the neutral condition (t = -0.21, p = 0.8373).

Interestingly, we found a high rank order correlation between groups for negative (r = 0.92, p < .001) and positive (r = 0.93, p < .001) self-beliefs. That is, participants in both groups chose very similar statements as self-relevant from the available pool. A list of the top and bottom five statements can be found in Supplement C, Table C.1 and Table C.2 in S1 File. As can be seen, frequency and consequently rank of the statements differ only slightly between groups. For example, in both groups, *“I hope I won’t embarrass myself”* was most frequently chosen as a self-relevant negative belief, chosen 90 times by HSA participants and 113 times by LSA participants. *“Even if things go badly for me*, *it is not a catastrophe*.*”*was most frequently chosen as a self-relevant positive belief, chosen by n = 97 HSA participants and n = 72 LSA participants.

### The role of agreement as a mediator

All estimation results for the two multilevel mediation models and a single level mediation model for comparison, are shown in Supplement D, Table D.1 in S1 File. For the multilevel models (M0; M1), the estimated mediated effects differ only marginally (Table D.1 in S1 File*)*. However, the two random intercepts model was superior to the one random intercept model for negative (χ2 = 37.80, df = 1, p < .001) and positive (χ2 = 122.90, df = 1, p < .001) self-beliefs, using the log-likelihood ratio test for nested models. We therefore report results of the two random intercepts model (M1).

#### Negative self-beliefs

**Fig 5:**
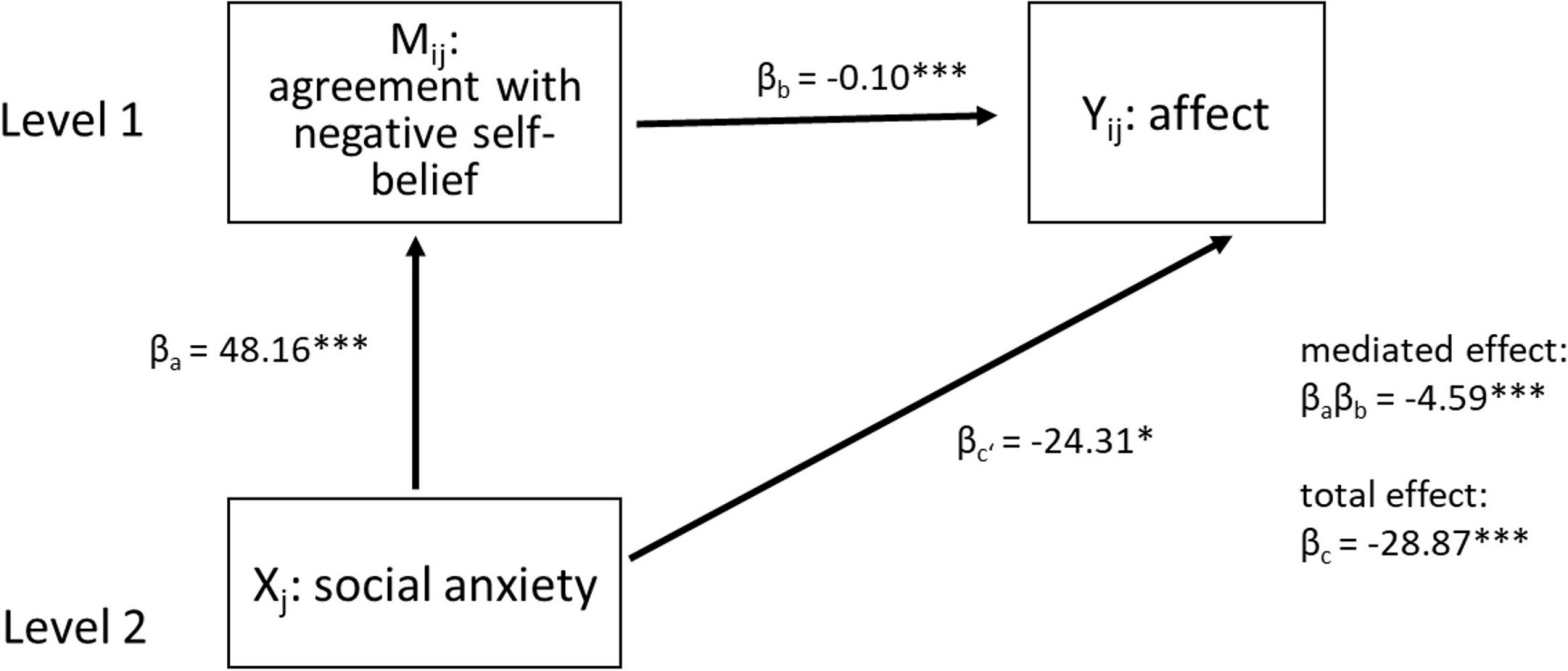
Estimation results of the mediation model for negative self-beliefs.

We found a significant negative direct effect (β_c’_ = -24.31, p < .05, 95% confidence interval (CI) [-43.25, -5.39]) of social anxiety (high vs. low) on affect as well as a significant positive effect on agreement (β_a_ = 48.16, p < .001, 95% CI [24.67, 71.65]). HSA participants reported more negative affect and higher agreement following negative self-beliefs than LSA participants. In addition, affect was negatively predicted by agreement with negative self-beliefs (β_b_ = -0.10, p < .001, 95% CI [-0.13, -0.07]). A significant proportion of the effect of social anxiety on affect was mediated by the agreement with negative self-beliefs (β_a_β_b_ = -4.59, p < .001). The proportion of the total effect of social anxiety on affect (β_c_ = -28.87, p < .01) explained by agreement was 12% with a 95% CI [6%, 40%]. Results are displayed in Fig 5.

#### Positive self-beliefs

**Fig 6:**
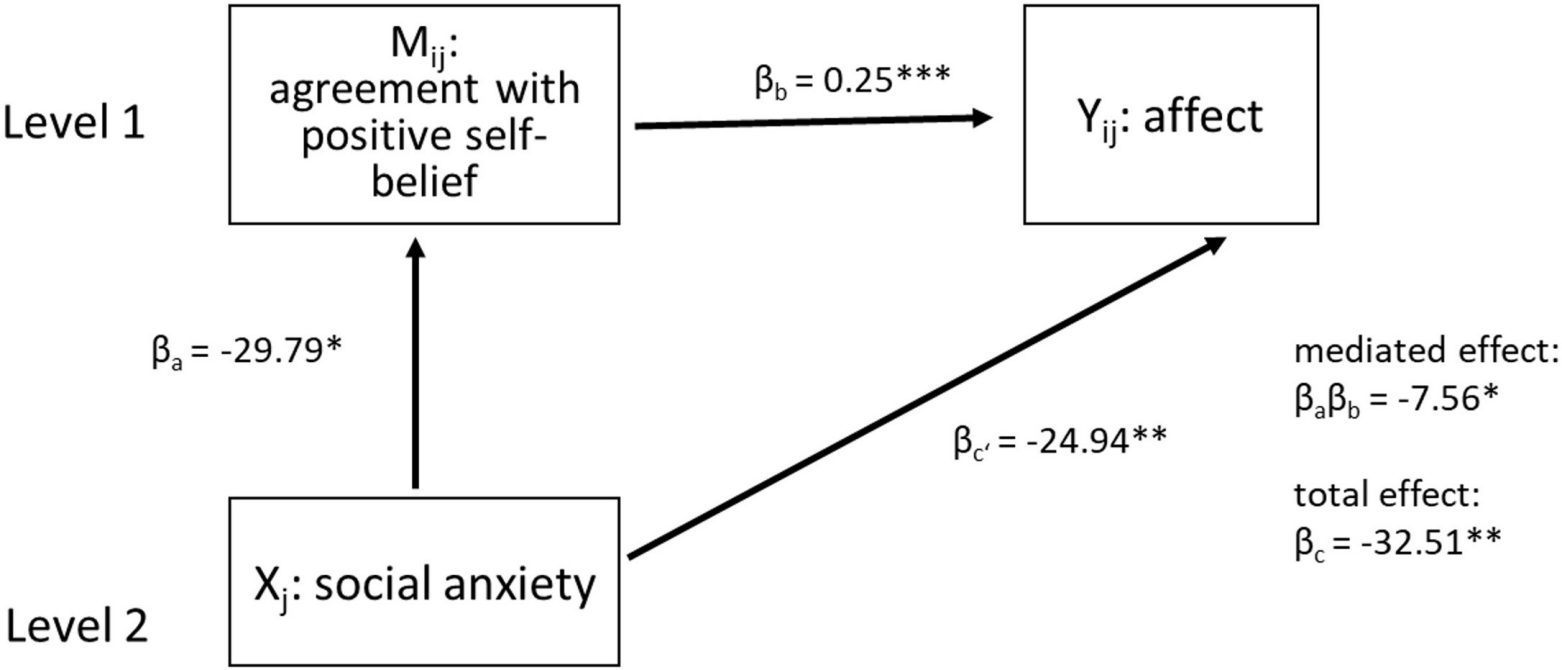
Estimation results of the mediation model for positive self-beliefs.

There is a significant direct negative effect (β_c’_ = -24.94, p < .01, 95% CI [-41.53, -8.37]) of social anxiety (high vs. low) on affect as well as on agreement with positive self-beliefs (β_a_ = -29.79, p < .05, 95% CI [-52.99, -6.59]). HSA participants reported less positive affect and less agreement following positive self-beliefs than LSA participants. In addition, affect is significantly (positively) predicted by agreement with positive self-beliefs (β_b_ = 0.25, p < 0.001, 95% CI [0.22, 0.29]). A significant proportion of the effect of social anxiety on affect is mediated by the agreement with positive self-beliefs (β_a_β_b_ = -7.56, p < .05). The proportion of the total effect of social anxiety on affect (β_c_ = -32.51, p < .01) explained by agreement is 21% with a 95% CI [0.06, 0.48]. Results are displayed in Fig 6.

## Discussion

Evidence on potential differences in the degree to which the individual is convinced of negative and positive self-beliefs has been largely missing in previous investigations of social anxiety and social anxiety disorder. In this study, we found that social anxiety increases affective responses towards negative and positive self-beliefs through an increase in agreeing with negative and decrease in agreeing with positive self-beliefs. Moreover, the content of self-beliefs that were chosen did not differ between low and high social anxiety. This indicates that high and low socially anxious individuals may in fact not differ strongly in the content of their thoughts, but in their appraisal of it. It might not be the fact that negative self-beliefs occur but rather the agreement with them (and potentially the frequency) that distinguishes high and low in social anxiety.

In line with previous findings [18, 22, 25], HSA participants agreed more with negative self-beliefs and less with positive self-beliefs compared to LSA participants. Our study extends these finding in the following ways. First, we found significant differences in agreement not only with negative, but also with positive self-beliefs. Second, we used semi-idiosyncratic beliefs that were associated with specific, individually feared social situations. Cognitive models of SAD indeed assume highly individualistic self-beliefs that are triggered bottom-up by certain situations. Our approach therefore corresponds more closely to these cognitive models and can therefore provide an important piece of empirical support for them. Third, we included an agreement rating as a direct measure of the strength of the beliefs. It is therefore possible to disentangle the content and valence (positive or negative) of, in addition to, the agreement with the belief. In addition, semi-idiosyncratic stimuli are more relevant to participants and can thus evoke emotional reactions that standardized stimuli sets cannot [42].

Mediation analysis revealed that the fact that HSA individuals displayed less positive affect might in part be explained by the fact that they agreed less with positive self-beliefs compared to LSA participants. We also showed that a substantial part of the negative affect that HSA participants experience in social situations, is mediated by the higher extent of agreement with negative self-beliefs. This supports the idea that not only the valence of a belief is relevant for the affective response but also the strength of the belief. We also found a high rank order correlation between HSA and LSA participants for positive as well as negative beliefs. The frequency with which each statement was chosen from our pre-selected item-pool was very similar between groups (s. Supplement B, Table B.1 and Table B.2 in S1 File*)*.

### Limitations and future directions

Affect ratings to individual self-beliefs within each block may not be independent from each other. For example, participants might (implicitly) anchor their ratings based on the negative self-belief presented first, resulting in an overall negativity bias for other ratings within this block. However, a block-wise design was used in order to create a realistic scenario and, thus, more ecologically valid task. In addition, although the order of presentation was the same for all participants, HSA participants still showed higher negativity following negative as well as positive self-beliefs compared to LSA participants. Each participant had an individual item-set, so we decided to keep the same order that the participants used when choosing their beliefs and adapted our statistical procedure. In addition, for each negative situation, we first presented a block of four negative self-beliefs. After a 6 s pause, we primed the same situation again and presented four positive self-beliefs. Lastly, neutral situations and beliefs were presented. Therefore, we cannot exclude that this fixed order might have caused a confounding effect.

We used an extreme group design, a-priori recruiting participants with either high or low social anxiety. Since the main focus of the current study was to test for the presence of relationships between social anxiety, self-beliefs and affect (rather than their strength), this approach seemed suitable despite recent criticism [43]. When used a-priori, it has been shown to provide more statistical power to find a true effect than an unselected sample with a comparable sample size [43–45].

We did not include a clinical sample of patients with diagnosed SAD for comparison. However, examining high socially vs. low socially anxious individuals, we could show that the agreement with (negative and positive) self-beliefs is significantly associated with affect in non-clinical individuals. That is, the observed effect likely applies to a large population varying in degrees of social anxiety rather than solely to patients diagnosed with SAD. The study could be replicated with a clinical SAD sample, testing whether the observed effects differ in people with a clinical diagnosis of SAD.

We did not assess how often positive or negative thoughts occur over a given time period in high vs. low socially anxious individuals. Frequency of positive self-beliefs might be lower in high socially anxious individuals. Future studies should investigate frequency and role of positive self-beliefs in more detail.

Our findings encourage the use of therapeutic interventions that do not only disconfirm negative self-beliefs that have previously been shown to be maladaptive by strengthening fear and avoidance. It might also be helpful to confirm more adaptive, positive self-beliefs. It is not only the content of a thought that can be disputed but also the extent to which the patient is convinced that this belief is true. In addition, these results provide a potential mechanism that could explain reported benefits of improving self-compassion in socially anxious individuals [e.g. 14–17]. Self-compassion is commonly defined as relating to the self with kindness and acceptance. It is possible that interventions that improve self-compassion lead to a higher a sense of conviction in positive self-beliefs.

## Conclusions

We established an experimental paradigm to assess how semi-idiosyncratic situation-specific negative and positive self-beliefs are related to affect in social anxiety. HSA participants reported higher negative and lower positive affect compared to LSA participants following exposure to individual negative and positive self-beliefs. HSA participants agreed more with negative and less with positive self-beliefs compared to LSA participants. Both groups selected similar beliefs as self-relevant. Results further indicate that the association between social anxiety and the affective response can be explained by the strength of the specific belief, for both positive and negative self-beliefs. Cognitive models of social anxiety could be improved by including not only the content of a belief but also the agreement with a belief. Practical implications include a stronger focus on confirmation of positive, functional self-beliefs in addition to disconfirming negative self-beliefs.

## Supporting information

S1 File Supplementary materials A, B, C, and D. Supplement A: Scales and questionnaires assessing positive and negative self-beliefs in social anxiety; Supplement B: Single level and multilevel model description for mediation analysis; Supplement C: List of top and bottom five sentences for high (HAS) and low (LSA) participants; Supplement D: Estimation results for the single-level and multilevel mediation models.
(DOCX)

## References

[1] Fehm L, Pelissolo A, Furmark T, Wittchen H-U. Size and burden of social phobia in Europe. European Neuropsychopharmacology. 2005;15: 453–462. doi: 10.1016/j.euroneuro.2005.04.002 15921898

[2] Jacobi F, Hofler M, Strehle J, Mack S, Gerschler A, Scholl L, et al. Psychische Störungen in der Allgemeinbevölkerung: Studie zur Gesundheit Erwachsener in Deutschland und ihr Zusatzmodul Psychische Gesundheit (DEGS1-MH). Der Nervenarzt. 2014;85: 77–87. doi: 10.1007/s00115-013-3961-y 24441882

[3] Fehm L, Beesdo K, Jacobi F, Fiedler A. Social anxiety disorder above and below the diagnostic threshold: prevalence, comorbidity and impairment in the general population. Social psychiatry and psychiatric epidemiology. 2008;43: 257–265. doi: 10.1007/s00127-007-0299-4 18084686

[4] Clark DM, Wells A. A cognitive model of social phobia. In: Heimberg R, Liebowitz M, Hope D, Schneider R, editors. Social phobia: Diagnosis, assessment and treatment. New York: Guilford Press; 1995. pp. 69–93.

[5] Heimberg R, Liebowitz M, Hope D, Schneider R, editors. Social phobia: Diagnosis, assessment and treatment. New York: Guilford Press; 1995.

[6] Rapee RM, Heimberg R. A cognitive–behavioral model of anxiety in social phobia. Behaviour research and therapy. 1995;35: 741–756. 10.1016/s0005-7967(97)00022-3 9256517

[7] Heimberg RC, Brozovich FA, Rapee RM. A cognitive behavioral model of social anxiety disorder: Update and extension. Social anxiety: Clinical, developmental, and social perspectives, 2nd ed. San Diego, CA, US: Elsevier Academic Press; 2010. pp. 395–422. doi: 10.1016/B978-0-12-375096-9.00015-8

[8] Radomsky AS, Alcolado GM, Abramowitz JS, Alonso P, Belloch A, Bouvard M, et al. Part 1—You can run but you can’t hide: Intrusive thoughts on six continents. Journal of Obsessive-Compulsive and Related Disorders. 2014;3: 269–279. doi: 10.1016/j.jocrd.2013.09.002

[9] Boden MT, John OP, Goldin PR, Werner K, Heimberg RG, Gross JJ. The role of maladaptive beliefs in cognitive-behavioral therapy: Evidence from social anxiety disorder. Behaviour research and therapy. 2012;50: 287–291. doi: 10.1016/j.brat.2012.02.007 22445947 PMC3327793

[10] Weeks JW, Howell AN. Fear of Positive Evaluation: The Neglected Fear Domain in Social Anxiety. In: Weeks JW, editor. The Wiley Blackwell Handbook of Social Anxiety Disorder. Chichester, West Sussex: Wiley-Blackwell; 2014. pp. 433–453.

[11] Glazier BL, Alden LE. Social anxiety disorder and memory for positive feedback. Journal of Abnormal Psychology. 2019;128: 228–233. doi: 10.1037/abn0000407 30702303

[12] Goldin PR, Ziv M, Jazaieri H, Weeks J, Heimberg RG, Gross JJ. Impact of cognitive-behavioral therapy for social anxiety disorder on the neural bases of emotional reactivity to and regulation of social evaluation. Behaviour Research and Therapy. 2014;62: 97–106. doi: 10.1016/j.brat.2014.08.005 25193002 PMC4253719

[13] Blackie RA, Kocovski NL. Examining the Relationships Among Self-Compassion, Social Anxiety, and Post-Event Processing. Psychol Rep. 2017;121: 669–689. doi: 10.1177/0033294117740138 29298554

[14] Blackie RA, Kocovski NL. Forgive and Let Go: Effect of Self-Compassion on Post-Event Processing in Social Anxiety. Mindfulness. 2018;9: 654–663. doi: 10.1007/s12671-017-0808-9

[15] Boersma K, Håkanson A, Salomonsson E, Johansson I. Compassion Focused Therapy to Counteract Shame, Self-Criticism and Isolation. A Replicated Single Case Experimental Study for Individuals With Social Anxiety. Journal of Contemporary Psychotherapy. 2015;45: 89–98. doi: 10.1007/s10879-014-9286-8

[16] Makadi E, Koszycki D. Exploring Connections Between Self-Compassion, Mindfulness, and Social Anxiety. Mindfulness. 2020;11: 480–492. doi: 10.1007/s12671-019-01270-z

[17] Stevenson J, Mattiske JK, Nixon RDV. The effect of a brief online self-compassion versus cognitive restructuring intervention on trait social anxiety. Behaviour Research and Therapy. 2019;123: 103492. doi: 10.1016/j.brat.2019.103492 31677528

[18] Goldin PR, Jazaieri H, Ziv M, Kraemer H, Heimberg R, Gross JJ. Changes in Positive Self-Views Mediate the Effect of Cognitive-Behavioral Therapy for Social Anxiety Disorder. Clinical psychological science: a journal of the Association for Psychological Science. 2013;1: 301–310. doi: 10.1177/2167702613476867 25541599 PMC4274797

[19] Thurston MD, Goldin P, Heimberg R, Gross JJ. Self-views in social anxiety disorder: The impact of CBT versus MBSR. Journal of anxiety disorders. 2017;47: 83–90. doi: 10.1016/j.janxdis.2017.01.001 28108059 PMC5376221

[20] Wong QJJ, Gregory B, Gaston JE, Rapee RM, Wilson JK, Abbott MJ. Development and validation of the Core Beliefs Questionnaire in a sample of individuals with social anxiety disorder. Journal of Affective Disorders. 2017;207: 121–127. doi: 10.1016/j.jad.2016.09.020 27721185

[21] Gregory B, Wong QJJ, Marker CD, Peters L. Maladaptive Self-Beliefs During Cognitive Behavioural Therapy for Social Anxiety Disorder: A Test of Temporal Precedence. Cognitive Therapy and Research. 2018;42: 261–272. doi: 10.1007/s10608-017-9882-5

[22] Gregory B, Peters L. Changes in the self during cognitive behavioural therapy for social anxiety disorder: A systematic review. Clinical psychology review. 2017;52: 1–18. doi: 10.1016/j.cpr.2016.11.008 27912159

[23] Helmes E, Holden RR, Ziegler M. Response Bias, Malingering, and Impression Management. In: Boyle GJ, Saklofske DH, Matthews G, editors. Measures of personality and social psychological constructs. London [etc.]: Academic Press; 2014. pp. 16–43. doi: 10.1016/B978-0-12-386915-9.00002-4

[24] Derry PA, Kuiper NA. Schematic processing and self-reference in clinical depression. Journal of Abnormal Psychology. 1981;90: 286–297. doi: 10.1037//0021-843x.90.4.286 7264058

[25] Goldin PR, Ziv M, Jazaieri H, Gross JJ. Randomized controlled trial of mindfulness-based stress reduction versus aerobic exercise: effects on the self-referential brain network in social anxiety disorder. Frontiers in human neuroscience. 2012;6: 295. doi: 10.3389/fnhum.2012.00295 23133411 PMC3488800

[26] Tanner RJ, Stopa L, De Houwer J. Implicit views of the self in social anxiety. Behaviour Research and Therapy. 2006;44: 1397–1409. doi: 10.1016/j.brat.2005.10.007 16337924

[27] Goldin PR, Manber-Ball T, Werner K, Heimberg R, Gross JJ. Neural mechanisms of cognitive reappraisal of negative self-beliefs in social anxiety disorder. Biological psychiatry. 2009;66: 1091–1099. doi: 10.1016/j.biopsych.2009.07.014 19717138 PMC2788040

[28] Hofmann SG, Moscovitch DA, Kim H-J, Taylor AN. Changes in self-perception during treatment of social phobia. Journal of consulting and clinical psychology. 2004;72: 588–596. doi: 10.1037/0022-006X.72.4.588 15301643

[29] Dryman MT, Heimberg RG. Emotion regulation in social anxiety and depression: a systematic review of expressive suppression and cognitive reappraisal. Clinical psychology review. 2018;65: 17–42. doi: 10.1016/j.cpr.2018.07.004 30064053

[30] Stangier U, Steffens M. Social Phobia Inventory (SPIN)—Deutsche Fassung. Frankfurt am Main: Psychologisches Institut der Universität Frankfurt am Main; 2002.

[31] Wittchen H-U, Zaudig M, Fydrich T. Strukturiertes Klinisches Interview für DSM-IV: Achse I und II. Göttingen: Hogrefe; 1997.

[32] Sosic Z, Gieler U, Stangier U. Screening for social phobia in medical in- and outpatients with the German version of the Social Phobia Inventory (SPIN). Journal of anxiety disorders. 2008;22: 849–859. doi: 10.1016/j.janxdis.2007.08.011 17923381

[33] Connor KM, Davidson JRT, Churchill LE, Sherwood A, Foa EB, Weisler RH. Psychometric properties of the Social Phobia Inventory (SPIN). The British Journal of Psychiatry. 2000;176: 379–386. 10827888 10.1192/bjp.176.4.379

[34] Clark DM. A Cognitive Perspective on Social Phobia. In: Crozier WR, Alden L, editors. International Handbook of Social Anxiety: Concepts, Research and Interventions Relating to the Self and Shyness. John Wiley & Sons; 2001. pp. 405–430.

[35] Lang PJ. Behavioral treatment and bio-behavioral assessment: computer applications. In: Sidowski JB, Johnson JH, Williams TA, editors. Technology in mental health care delivery systems. Norwood, NJ: Ablex; 1980. pp. 119–137.

[36] Suk H-J. Color and Emotion—a study on the affective judgment across media and in relation to visual stimuli. University of Mannheim: Dissertaion; 2006.

[37] Bates D, Mächler M, Bolker B, Walker S. Fitting Linear Mixed-Effects Models Using lme4. Journal of Statistical Software. 2015;67. doi: 10.18637/jss.v067.i01

[38] Tingley D, Yamamoto T, Hirose K, Keele L, Imai K. mediation: R Package for Causal Mediation Analysis. Journal of Statistical Software. 2014;59.

[39] Krull JL, MacKinnon DP. Multilevel Modeling of Individual and Group Level Mediated Effects. Multivariate behavioral research. 2001;36: 249–277. doi: 10.1207/S15327906MBR3602_06 26822111

[40] Preacher KJ, Zyphur MJ, Zhang Z. A general multilevel SEM framework for assessing multilevel mediation. Psychological methods. 2010;15: 209–233. doi: 10.1037/a0020141 20822249

[41] Imai K, Keele L, Tingley D. A general approach to causal mediation analysis. Psychological methods. 2010;15: 309–334. doi: 10.1037/a0020761 20954780

[42] Viol K, Aas B, Kastinger A, Kronbichler M, Schöller H, Reiter E-M, et al. Individual OCD-provoking stimuli activate disorder-related and self-related neuronal networks in fMRI. Psychiatry research Neuroimaging. 2019;283: 135–144. doi: 10.1016/j.pscychresns.2018.12.008 30594423

[43] Fisher JE, Guha A, Heller W, Miller GA. Extreme-groups designs in studies of dimensional phenomena: Advantages, caveats, and recommendations. Journal of Abnormal Psychology. 2020;129: 14–20. doi: 10.1037/abn0000480 31657600 PMC6928394

[44] Feldt LS. The use of extreme groups to test for the presence of a relationship. Psychometrika. 1961;26: 307–316. doi: 10.1007/BF02289799

[45] Fowler RL. Using the Extreme Groups Strategy When Measures Are Not Normally Distributed. Applied Psychological Measurement. 1992;16: 249–259. doi: 10.1177/014662169201600305

