# Supplementary material for: Negative and positive self-beliefs in social anxiety: The strength of believing mediates the affective response
Source: PLoS One. 2023 Mar 15;18(3):e0281387. doi: 10.1371/journal.pone.0281387 (PMC10016721; doi:10.1371/journal.pone.0281387)
Supplement: S1 File — Supplementary materials A, B, C, and D. Supplement A: Scales and questionnaires assessing positive and negative self-beliefs in social anxiety; Supplement B: Single level and multilevel model description for mediation analysis; Supplement C: List of top and bottom five sentences for high (HAS) and low (LSA) participants; Supplement D: Estimation results for the single-level and multilevel mediation models. (DOCX) [file pone.0281387.s001.docx]

**Supplementary Material**

Supplement A: Scales and questionnaires assessing positive and negative self-beliefs in social anxiety.

Additional items assessing positive self-beliefs were constructed by reformulating negative self-beliefs.

*Table A.1*

Overview of scales assessing negative self-beliefs in social anxiety

| **negative self-beliefs** | **n Items selected** |
| --- | --- |
| Self-Statements during Public Speaking Questionnaire, SSPS [1,2] | 5 |
| Social Cognitions Questionnaire, SCQ [3,4] | 22 |
| Social Attitudes Questionnaire, SAQ [5,6] | 38 |
| Social Interaction Self-Statement Test, SISST [7] | 15 |
| Brief Fear of Negative Evaluation Scale, FNEB [8,9] | 12 |
| Social Thoughts and Beliefs Scale, STABS [10] | 21 |
| Maladaptive Interpersonal Belief Scale, MIBS [11] | 4 |
| The Negative Self-Portrayal Scale, NSPS [12] | 23 |
| Core Belief Questionnaire, CBQ [13] | 17 |
| **total** | **157** |

*Table A.2*

Overview of scales assessing positive self-beliefs in social anxiety

| **positive self-beliefs** | **n Items selected** |
| --- | --- |
| Social Attitudes Questionnaire, SAQ [5,6] | 9 |
| Social Interaction Self-Statement Test, SISST [7] | 15 |
| Maladaptive Interpersonal Belief Scale, MIBS [11] | 5 |
| Automatic Thoughts Questionnaire-Revised, ATQ-R [14,15] | 5 |
| **total** | **34** |

Supplement B: Single level and multilevel model description for mediation analysis

*Reasoning for multilevel instead of single level modeling approach*

We used a multilevel mediation modeling approach. There were high ICCs (e.g. agreement with positive self-beliefs ICC = 0.38; agreement with negative self-beliefs ICC = 0.35) as they are common in repeated measurement designs [16] indicating high interdependency. Violation of independent observations in ordinary least square estimation (OLS) has been shown to result in downwardly biased standard error estimation, large test statistics and subsequently inflated Type I error rates [17]. Since multilevel modeling was developed to handle these challenges, we chose to employ multilevel instead of single-level mediation modeling.

*Multilevel mediation method used for M1 and M0*

For the model M1, we used the traditional multilevel mediation method described by Krull and MacKinnon [17] for a 2-1-1 design, because it can easily be applied to a two random intercepts model. This approach translates single level regression models for the direct and indirect effect into a multilevel structure. The product of the estimated effects $\hat{\text{β}}$a$\hat{\text{β}}$b is used to estimate the mediated effect and Zobel’s test is used to estimate standard errors [17]. P-values were retrieved from <http://quantpsy.org/sobel/sobel.html>.

For the model M0, we used the “mediate” function from the R package “mediation” [18] to conduct a second mediation analysis for the basic model (M0) to double check our results. The “mediate” package relies on the counterfactual framework, which is a widely used approach in the statistical sciences. According to this approach, the average causal mediation effects are non-parametrically identified under the sequential ignorability assumption. Thus, they can be estimated without any additional assumptions regarding distribution or functional form [19]. The approach can be used in multilevel mediation models [18,20]. For a detailed description of the statistical theory and procedures underlying the package see [18,19].

*Table B.1* shows the model equations for a single level mediation model, the basic random intercept model (M0) and the alternative two random intercept model (M1).

Table B.1

Single level and multilevel model equations for mediation analysis

| Single Level | Multilevel | |
| --- | --- | --- |
|  | M0: random intercept only | M1: two random intercepts (u_oi_) |
| Outcome  Y*_ij_* = β*_0_* + β*_c‘_*X*_j_* + β*_b_*M*_ij_* + r*_ij_*  Mediator  M*_ij_* = β*_0_* + β*_a_*X*_j_* + r*_ij_* | Outcome  Level 1: Y*_ij_* = β*_0j_* + β*_b_*M*_ij_* + r*_ij_*  Level 2: β*_0j_* = γ*_00_* + γ*_c‘_*X*_j_* + u*_oj_*  total: Y*_ij_* = γ*_00_* + γ*_c‘_*X*_j_* + β*_b_*M*_ij_* +  u*_oj_* + r*_ij_*  Mediator  Level 1: M*_ij_* = β*_0j_* + r*_ij_*  Level 2: β*_0j_* = γ*_00_* + γ*_a_*X*_j_* + u*_oj_*  total: Y*_ij_* = γ*_00_* + γ*_a_*X*_j_* + u*_oj_* + r*_ij_* | Outcome  Level 1: Y*_ij_* = β*_0j_* + β*_b_*M*_ij_* + r*_ij_*  Level 2: β*_0j_* = γ*_00_* + γ*_c‘_*X*_j_* + u*_oj_* + u*_oi_*  total: Y*_ij_* = γ*_00_* + γ*_c‘_*X*_j_* + β*_b_*M*_ij_* + u*_oj_*  + u*_oi_* + r*_ij_*  Mediator  Level 1: M*_ij_* = β*_0j_* + r*_ij_*  Level 2: β*_0j_* = γ*_00_* + γ*_a_*X*_j_* + u*_oj_* + u*_oi_*  total: Y*_ij_* = γ*_00_* + γ*_a_*X*_j_* + u*_oj_* + u*_oi_* + r*_ij_* |

*Note:* Y = subjective affective rating (outcome), M = agreement (mediator), X = group (high socially anxious vs. low socially anxious) (predictor)*.*

Supplement C: List of top and bottom five sentences for high (HSA) and low (LSA) participants

*Table C.1* and *Table C.2* show the five most and five less frequently chosen negative and positive self-beliefs for HSA and LSA. As can be seen, there are only slight differences in frequency and rank between groups.

*Table C.1*

Frequency and rank of negative self-beliefs

|  | HSA | | LSA | |
| --- | --- | --- | --- | --- |
| rank | belief | n | belief | n |
| 1 | I hope I won’t embarrass myself. | 90 | I hope I won’t embarrass myself. | 113 |
| 2 | People will see that I am nervous. | 69 | People will see that I am nervous. | 90 |
| 3 | What I am going to say will sound stupid. | 67 | What I am going to say will sound stupid. | 78 |
| 4 | People will stare at me. | 62 | People will stare at me. | 76 |
| 5 | Others find my behavior inadequate. | 61 | Others find my behavior inadequate. | 61 |
| … |  |  |  |  |
| 25 | I am embarrassed if others see me sweating. | 15 | Others think I am unimportant. | 12 |
| 26 | I am unattractive. | 12 | Others notice that I am boring. | 12 |
| 27 | It is my fault if others don’t like me. | 10 | It is my fault if others don’t like me. | 8 |
| 28 | I am not lovable. | 6 | I am not lovable. | 6 |
| 29 | I am a coward when I am with others. | 4 | I am a coward when I am with others. | 4 |

*Note:* n = frequency, HSA = high socially anxious, LSA = low socially anxious.

*Table C.2*

Frequency and rank of positive self-beliefs

|  | HSA | | LSA | |
| --- | --- | --- | --- | --- |
| rank | belief | n | belief | n |
| 1 | Even if things go badly for me, it is not a catastrophe. | 97 | Even if things are unpleasant, I can deal with it. | 72 |
| 2 | Even if things are unpleasant, I can deal with it. | 80 | Even if things go badly for me, it is not a catastrophe. | 70 |
| 3 | What do I have to lose? It is worth a try. | 74 | What do I have to lose? It is worth a try. | 63 |
| 4 | I hope I am going to make a good impression. | 72 | I don’t have to be liked by everyone. | 61 |
| 5 | I don’t have to be liked by everyone. | 70 | I am as competent as others. | 51 |
| … |  |  |  |  |
| 31 | Others like me. | 7 | Others think I am interesting. | 10 |
| 32 | I am valuable to other people. | 6 | I am valuable to other people. | 10 |
| 33 | Others think I am interesting. | 5 | I am valuable. | 10 |
| 34 | I am good at small talk. | 4 | I am attractive. | 7 |
| 35 | I am valuable. | 4 | I am good at small talk. | 5 |

*Note:* n = frequency, HSA = high socially anxious, LSA = low socially anxious.

Supplement D: Estimation results for the single-level and multilevel mediation models

*Table D.1* shows the complete results for all three different models (single level, M0, M1) for comparison. Estimates of the two random intercept models are also illustrated by Figure 4 and Figure 5 in the manuscript.

*Table D.1*

Single-level and multilevel estimates (and standard errors) of β_a,_ β_b_, and the mediated effect β_a_β_b_ for negative and positive self-beliefs

|  | Analysis Type | | | | | |
| --- | --- | --- | --- | --- | --- | --- |
|  | Single-Level | | | Multilevel: M0, *M1* | | |
| Negative self-beliefs | | | |  | | |
| Estimate | β_a_ | β_b_ | β_a_β_b_ | β_a_ | β_b_ | β_a_β_b_ |
| Agreement (Mediator) | -46.78*** (11.94) | -0.32*** (0.08) | 15.17**  (5.42) | *M_0_: -45.88*  *t = -3.829,*  *M_1_:* -48.16***  (11.97) | *M_0_: -0.08*  *t = -5.312,*  *M_1_:* -0.10***  (0.02) | *M_0_: 3.55***,*  M_1_: 4.59***  (1.36) |
| Estimate | β_c_ | β_c’_ |  | β_c_ | β_c’_ |  |
| Social anxiety (X) | 29.56***  (10.24) | 14.40  (10.25) |  | *M_0_: 29.02***,* M_1_: 28.87** (10.02) | *M_0_: 25.10****  M_1_: 24.31* (9.65) |  |
| Positive self-beliefs | | | |  | | |
| Estimate | β_a_ | β_b_ | β_a_β_b_ | β_a_ | β_b_ | β_a_β_b_ |
| Agreement  (Mediator) | 30.24*  (12.24) | 0.48***  (0.07) | 14.38*  (6.16) | *M_0_: 28.74*  *t = 2.408,*  *M_1_:* 29.79*  (11.83) | *M_0_: 0.25*  *t = 14.49,*  *M_1_:* 0.25***  (0.01) | *M_0_: 7.62*^2^*  *M_1_ :* 7.56*  (3.04) |
| Estimate | β_c_ | β_c’_ |  | β_c_ | β_c’_ |  |
| Social anxiety (X) | 36.62***  (9.94) | 22.25**  (8.35) |  | *M_0_: 33.02**** M_1_: 32.51**  (9.88) | *M_0_: 26.38****  M_1_: 24.94**  (8.45) |  |

*Note:* M0 = one random intercept model, estimation following Tingley et al. 2014 [18], note that p-values are not supplied for direct effects on the mediator (β_a_) or direct effects of the mediator on the outcome (β_b_) following this approach; M1 = two random intercepts model, estimation following Krull & MacKinnon, 2001 [17]; p< 0.05*, p< 0.01**, p < 0.001***.

**References**

1. Arend, Matthias G., and Thomas Schäfer. 21. Gerlach AL, Heinrichs N, Bandl C, Zimmermann T. SSPS - Ein Fragebogen zur Erfassung der kognitiven Komponente von Redeangst. Zeitschrift für Klinische Psychologie und Psychotherapie. 2007;36: 112–120. doi:10.1026/1616-3443.36.2.112

2. Hofmann SG, DiBartolo PM. An Instrument to Assess Self-Statements During Public Speaking: Scale Development and Preliminary Psychometric Properties. Behavior Therapy. 2000;31: 499–515. doi: 10.1016/S0005-7894(00)80027-1

3. Wells A, Stopa LA, Clark DM. Social cognitions questionnaire. Unpublished Manuscript. Department of Psychiatry, Oxford University, Oxford, UK; 1993.

4. Stangier U, Heidenreich T, Ehlers A, Clark D. Fragebogen zu sozialphobischen Kognitionen (SPK) (Unveröffentlichtes Manuskript). Johann Wolfgang Goethe-Universität, Frankfurt; 1996.

5. Clark DM. Social attitudes questionnaire. Unpublished manuscript. Department of Psychiatry, Oxford University, Oxford, UK; 1995.

6. Hoyer J, Margraf J. Angstdiagnostik: Grundlagen und Testverfahren. Springer-Verlag; 2013.

7. Glass CR, v. Merluzzi T, Biever JL, Larsen KH. Cognitive assessment of social anxiety: Development and validation of a self-statement questionnaire. Cognitive Therapy and Research. 1982;6: 37–55. doi:10.1007/BF01185725

8. Collins KA, Westra HA, Dozois DJA, Stewart SH. The validity of the brief version of the Fear of Negative Evaluation Scale. Journal of Anxiety Disorders. 2005;19: 345–359. doi:10.1016/j.janxdis.2004.02.003

9. Reichenberger J, Schwarz M, König D, Wilhelm FH, Voderholzer U, Hillert A, et al. Angst vor negativer sozialer Bewertung: Übersetzung und Validierung der Furcht vor negativer Evaluation–Kurzskala (FNE-K). Diagnostica. 2016;62: 169–181. doi:10.1026/0012-1924/a000148

10. Turner SM, Johnson MR, Beidel DC, Heiser NA, Lydiard RB. The Social Thoughts and Beliefs Scale: a new inventory for assessing cognitions in social phobia. Psychological assessment. 2003;15: 384–391. doi:10.1037/1040-3590.15.3.384

11. Boden MT, John OP, Goldin PR, Werner K, Heimberg RG, Gross JJ. The role of maladaptive beliefs in cognitive-behavioral therapy: Evidence from social anxiety disorder. Behaviour research and therapy. 2012;50: 287–291. doi:10.1016/j.brat.2012.02.007

12. Moscovitch DA, Huyder V. The negative self-portrayal scale: development, validation, and application to social anxiety. Behavior Therapy. 2011;42: 183–196. doi:10.1016/j.beth.2010.04.007

13. Wong QJJ, Gregory B, Gaston JE, Rapee RM, Wilson JK, Abbott MJ. Development and validation of the Core Beliefs Questionnaire in a sample of individuals with social anxiety disorder. Journal of Affective Disorders. 2017;207: 121–127. doi:10.1016/j.jad.2016.09.020

14. Kendall PC, Howard BL, Hays RC. Self-referent speech and psychopathology: The balance of positive and negative thinking. Cognitive Therapy and Research. 1989;13: 583–598. doi:10.1007/BF01176069

15. Pössel P, Seemann S, Hautzinger M. Evaluation eines deutschsprachigen Instrumentes zur Erfassung positiver und negativer automatischer Gedanken. Zeitschrift für Klinische Psychologie und Psychotherapie. 2005;34: 27–34. doi:10.1026/1616-3443.34.1.27

16. Arend MG, Schäfer T. Statistical power in two-level models: A tutorial based on Monte Carlo simulation. Psychological methods. 2019;24: 1–19. doi:10.1037/met0000195

17. Krull JL, MacKinnon DP. Multilevel Modeling of Individual and Group Level Mediated Effects. Multivariate behavioral research. 2001;36: 249–277. doi:10.1207/S15327906MBR3602

18. Tingley D, Yamamoto T, Hirose K, Keele L, Imai K. mediation: R Package for Causal Mediation Analysis. Journal of Statistical Software. 2014;59. doi: 10.18637/jss.v059.i05

19. Imai K, Keele L, Tingley D. A general approach to causal mediation analysis. Psychological methods. 2010;15: 309–334. doi:10.1037/a0020761

20. Sales AC. Review: mediation Package in R. Journal of Educational and Behavioral Statistics. 2017;42: 69–84. doi:10.3102/1076998616670371
